# Causal association between obstructive sleep apnea and gastroesophageal reflux disease: A bidirectional two-sample Mendelian randomization study

**DOI:** 10.3389/fgene.2023.1111144

**Published:** 2023-04-05

**Authors:** Qianyin Zhu, Lijiangshan Hua, Lingshan Chen, Tingyu Mu, Die Dong, Jiayi Xu, Cuizhen Shen

**Affiliations:** School of Nursing, Zhejiang Chinese Medical University, Hangzhou, Zhejiang, China

**Keywords:** Mendelian randomization, obstructive sleep apnea, gastroesophageal reflux disease, bidirectional, causal

## Abstract

**Objectives:** Correlations between obstructive sleep apnea (OSA) and gastroesophageal reflux disease (GERD) have been detected in previous observational studies. However, this association remains uncertain due to the potential presence of selection and confounding biases. Therefore, this bidirectional two-sample Mendelian randomization (MR) study was conducted to evaluate the causal relationship between OSA and GERD.

**Methods:** In this study, instrumental variables (IVs) for OSA were selected from publicly available genetic summary data (27,207 cases and 280,720 controls). Summary statistics for GERD were obtained from a genome-wide association study of 602,604 individuals. The inverse variance weighted (IVW) method was used as the main MR method. The MR-Egger intercept test, MR pleiotropy residual sum and outlier, and leave-one-out analysis were used to detect pleiotropy. Heterogeneity was detected by Cochran’s Q test.

**Results:** The IVW results revealed that OSA [odds ratio (OR): 1.19, 95% confidence interval (CI): 1.11–1.28, *p* = 8.88E-07] was causally associated with the incidence of GERD. Moreover, there was evidence of GERD leading to OSA in the IVW analysis (OR: 1.44, 95%CI: 1.33–1.57, *p* = 7.74E-19). No directional pleiotropy was detected by the MR-Egger intercept test (all *p* > 0.05).

**Conclusion:** This study found that OSA is linked to a higher incidence of GERD, and *vice versa*. This finding might be helpful for the screening and prevention of these two diseases.

## 1 Introduction

Obstructive sleep apnea (OSA) is a prevalent disease characterised by disordered breathing during sleep. Specifically, in OSA, temporary upper airway stricture during sleep causes sleep deficiency, intermittent hypoxia and autonomic fluctuation (Patel, 2019). Epidemiological studies have estimated that approximately 22% of men and 17% of women have OSA (Franklin and Lindberg., 2015). Because of its troublesome symptoms and chronic course, OSA significantly reduces a patient’s health-related quality of life (Gottlieb and Punjabi., 2020). Gastroesophageal reflux disease (GERD) is a functional gastrointestinal disorder characterized by heartburn and regurgitation (Katz et al., 2022). According to current epidemiological research, the prevalence of GERD has increased by more than 77% over the past 10 years (Zhang D. et al., 2022). The increasing prevalence of GERD has resulted in increasingly high healthcare expenditure (Howden et al., 2021). Previous studies have identified a bidirectional relationship between OSA and GERD, where GERD appears to increase the risk of OSA and, on the other hand, OSA contributes to the development of GERD (Shibli et al., 2020).

A nationwide study reported that 12.21% of GERD patients have a concurrent diagnosis of OSA, with the prevalence being approximately triple that observed in the general population (Mahfouz et al., 2022). Similarly, compared to people without OSA, nocturnal gastroesophageal reflux is more common in OSA patients (Fass et al., 2005). In addition, continuous positive airway pressure treatment can reduce gastroesophageal reflux alongside improvements in OSA symptoms (Wang et al., 2020). A cross-sectional study observed a positive correlation between GERD and OSA, and this relationship was unlikely to be affected by potential confounders and different diagnostic criteria (Gilani et al., 2016). However, several studies have produced conflicting results. From example, Shepherd et al. found that obesity, rather than OSA *per se*, is the cause of GERD in OSA patients (Shepherd and Orr., 2016). Another study reported that the occurrence of GERD had no relation to the severity of OSA (Kuribayashi et al., 2010). Thus, the association between OSA and GERD remains controversial.

Mendelian randomization (MR) is a data analysis method that uses genotypes as instrumental variables (IVs) to infer causal relationships between features of interest (Xu et al., 2022). Mendel’s second law states that genotypes are allocated randomly at conception and are unlikely to be affected by environmental confounders. In addition, DNA sequences are invariant, and the flow of biological information is unidirectional. Therefore, MR can minimize reverse causation and avoid confusion (Swerdlow et al., 2016). Two-sample MR is an extension of the basic MR design. This MR analysis is conducted using the exposure and outcome data obtained from different or partially overlapping samples (Hartwig et al., 2016). It is widely applicable and can avoid pseudo-positive results. Therefore, this study implemented a two-sample MR analysis to reveal the causal relationship between OSA and GERD. The data for this analysis were obtained from published genome-wide association studies (GWAS).

## 2 Materials and methods

### 2.1 Study design

**FIGURE 1:**
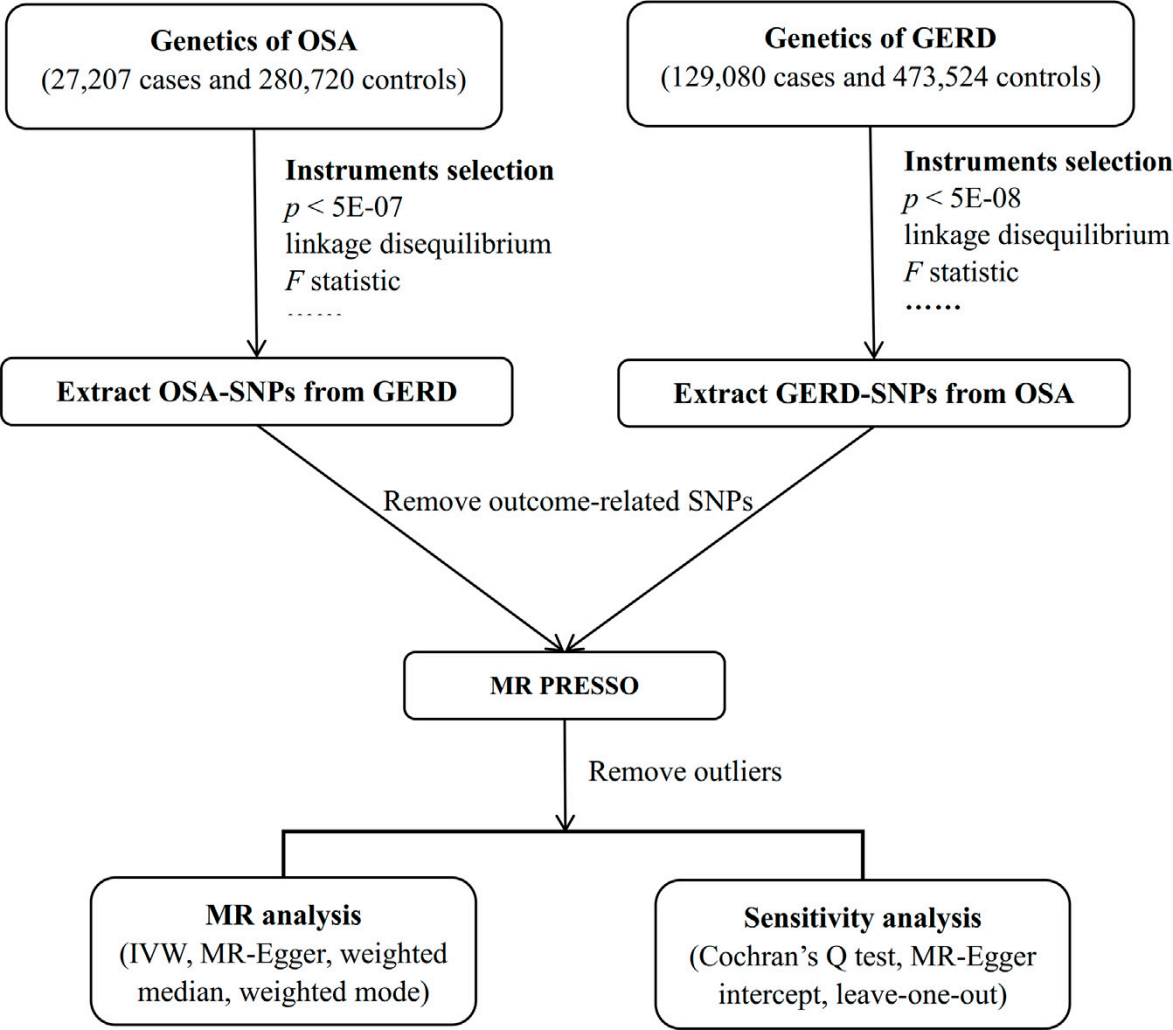
Workflow of the bidirectional two-sample MR study. Abbreviation: MR, Mendelian randomization; OSA, obstructive sleep apnea; GERD, gastroesophageal reflux disease; SNP, single nucleotide polymorphism; PRESSO, Pleiotropy REsidual Sum and Outlier; IVW, inverse variance weighted.

Because the data were collected through publicly available online databases, ethical approval and informed consent were not applicable. The MR design flowchart of the current study is presented in Figure 1. For MR, the selected IVs must satisfy the following assumptions: 1) correlation: IVs must be strongly correlated with exposure; 2) exclusive: IVs should not directly affect the outcome; 3) independence: IVs should not affect the outcome through any confounding variables (Emdin et al., 2017). The “*TwoSampleMR*” package (version 0.5.6) and the “*MRPRESSO*” package (version 1.0) in R (version 4.2.1) were used to perform the statistical analyses.

### 2.2 Data source

Candidate genetic instruments for OSA were extracted from the FinnGen database, which consists of 27,207 cases and 280,720 controls. These data can be found at https://r7.finngen.fi (Kurki et al., 2022). All subjects in the FinnGen data were of European ancestry.

Summary statistics for GERD phenotypes were obtained from the IEU GWAS database (https://gwas.mrcieu.ac.uk/datasets/ebi-a-GCST90000514/). This database comprises 602,604 European ancestry participants, including 129,080 GERD cases and 473,524 control cases (Ong et al., 2022) (Supplementary Table S1).

### 2.3 Selection of instrumental variables

For the selection of eligible IVs, single nucleotide polymorphisms (SNPs) should meet various requirements. First, SNPs should be associated with the exposure at the genome-wide significance level (*p* < 5E-08). However, in this MR analysis, a more relaxed threshold (*p* < 5E-07) was used for OSA in order to include more SNPs; such a threshold has been used previously in many MR studies (Zhang Y. et al., 2022). Second, the PLINK algorithm was used to obtain SNPs that were not in linkage disequilibrium (*r* ^2^ <0.001; clumping window size = 10,000 kb). Third, manual selection was performed in the PhenoScanner GWAS database (http://phenoscanner.medschl.cam.ac.uk) to exclude outcome trait-related IVs. Fourth, SNPs with an *F* statistic <10 were regarded as weak IVs and were excluded (Pierce et al., 2011). The calculation formula for the *F* statistic is *F* = *R* ^2^(N-2)/(1-*R* ^2^), where N is the sample size and *R* ^2^ reflects the proportion of variance explained by the SNPs. *R* ^*2*^ was calculated as 2 × MAF × (1-MAF) × beta^2^, where MAF is the minor allele frequency of the SNP and beta is the effect size of the SNP. Finally, palindromic SNPs and SNPs associated with the outcome at the genome-wide significance level were discarded.

### 2.4 Mendelian randomization analyses

In this study, inverse variance weighted (IVW), MR-Egger, weighted median and weighted mode were applied to investigate the causal relationship between OSA and GERD. IVW assumes that all SNPs are valid IVs. It has higher statistical power than other MR methods, and thus, IVW was used as the main analysis in this study (Lin et al., 2021). Unless otherwise specified, random-effects IVW was performed because the causal inference of this method is more conservative and takes into account uncertainty due to pleiotropy (Bowden et al., 2018). MR-Egger, weighted median and weighted mode were implemented as complementary methods to IVW; these have different underlying assumptions for horizontal pleiotropy and provide more robust MR estimates in broader scenarios. When at least half of the IVs are invalid, the causal relationship can also be estimated consistently by the weighted median (Bowden et al., 2016). MR-Egger considers the existence of an intercept term in weighted linear regression. It does not strictly require an absence of pleiotropy between genetic variants, which is in contrast to the IVW method (Bowden et al., 2015). The weighted mode method can evaluate the causal relationship between the outcome and a subset of SNPs by grouping the SNPs into subgroups based on similarities in their causal effects. However, compared to the IVW and weighted median methods, it is less able to detect a causal effect (Hartwig et al., 2017).

### 2.5 Sensitivity analyses

Sensitivity analyses were conducted to determine the robustness of the MR results. The heterogeneity test (Cochran’s Q test in the IVW method) was used to examine the differences between each IV; *p* < 0.05 was taken to represent the presence of heterogeneity. MR Pleiotropy RESidual Sum and Outlier (PRESSO) was used to detect horizontal pleiotropy and identify outlier variants. After removing SNP outliers, an outlier-corrected MR analysis was conducted to obtain an unbiased causal estimate (Verbanck et al., 2018). Directional pleiotropy was also appraised by the MR-Egger intercept test; a non-zero intercept suggests that the set of IVs suffers from directional pleiotropy (Burgess and Thompson., 2017). In addition, leave-one-out analysis was performed to evaluate whether the MR results were strongly driven by a specific SNP.

## 3 Results

### 3.1 Effect of obstructive sleep apnea on gastroesophageal reflux disease

**FIGURE 2:**
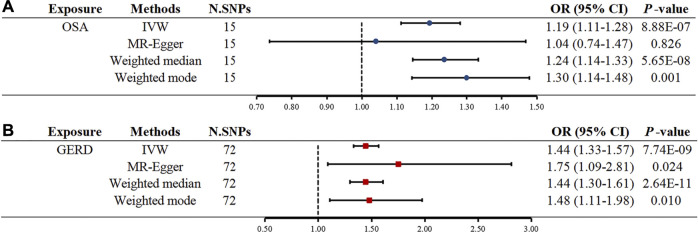
Two-sample MR estimates results of causal associations between OSA and GERD. **(A)** Causal estimates result for OSA on GERD. **(B)** Causal estimates result for GERD on OSA. Abbreviation: MR, Mendelian randomization; OSA, obstructive sleep apnea; GERD, gastroesophageal reflux disease; IVW, inverse variance weighted; N. SNPs is the number of SNPs after outliers removed; SNPs, single nucleotide polymorphisms; OR, odds ratio; CI, confidence interval.

**FIGURE 3:**
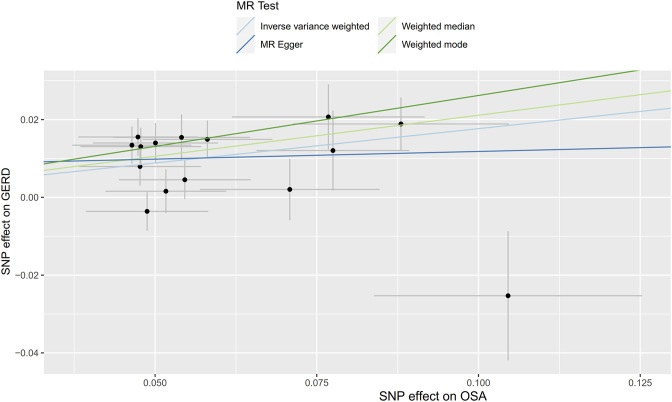
Scatter plot for the causal relationship of OSA on GERD. MR, Mendelian randomization; SNP, single nucleotide polymorphism; OSA, obstructive sleep apnea; GERD, gastroesophageal reflux disease.

**FIGURE 4:**
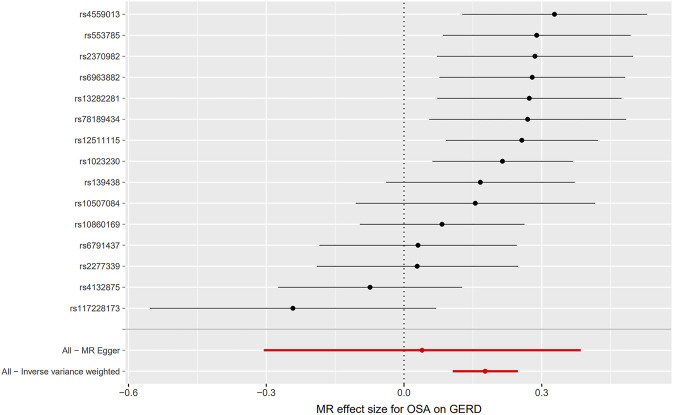
Forest plot for the causal relationship of OSA on GERD. MR, Mendelian randomization; OSA, obstructive sleep apnea; GERD, gastroesophageal reflux disease.

Initially, SNPs associated with OSA at the genome-wide significance level and those that were not in linkage disequilibrium were identified. After excluding palindromic SNPs and GERD-related SNPs, 21 SNPs were attained as IVs; these explain 3% of the phenotypic variation. The *F* statistic of each IV ranged from 325 to 492, indicating that all IVs were of good strength (all *F* >10) (Supplementary Tables S2). However, MR PRESSO indicated that rs10125995, rs10917318, rs11030323, rs2529273, rs76229479, and rs742760 were pleiotropic SNPs. After removing the SNP outliers, MR analysis was applied to evaluate the relationship between OSA and GERD. The IVW results [odds ratio (OR): 1.19, 95% confidence interval (CI): 1.11–1.28, *p* = 8.88E-07], weighted median results (OR: 1.24, 95% CI: 1.14–1.33, *p* = 5.65E-08) and weighted mode results (OR: 1.30, 95% CI: 1.14–1.48, *p* = 0.001) suggested that OSA was causally associated with the incidence of GERD. However, the MR-Egger results did not provide evidence of a causal association between OSA and GERD (OR: 1.04, 95% CI: 0.74–1.47, *p* = 0.826) (Figure 2A). This is probably because the MR-Egger method has lower causal estimate power than the other MR methods (Burgess and Thompson., 2017). The scatter plot and forest plot for these analyses are presented in Figures 3, 4. Heterogeneity was observed by Cochran’s Q test (Q = 25.75, *p* = 0.028). The MR-Egger intercept analysis results revealed no directional pleiotropy (*p* = 0.439) (Table 1). In the leave-one-out analysis, no single SNP strongly influenced the overall effect of OSA on GERD (Supplementary Figure S1).

**TABLE 1:** Heterogeneity and horizontal pleiotropy analyses between OSA and GERD.

Exposure	Outcome	MR PRESSO (after outliers removed)		MR-egger intercept		Heterogeneity	
		RSSobs	*p*-value	Intercept	*p*-intercept	Cochran’s Q	Q-pval
OSA	GERD	28.99	0.060	0.008	0.439	25.75	0.028
GERD	OSA	94.49	0.073	−0.006	0.423	91.75	0.049

Abbreviation: OSA, obstructive sleep apnea; GERD, gastroesophageal reflux disease; MR, Mendelian randomization; PRESSO, pleiotropy RESidual sum and outlier.

### 3.2 Effect of gastroesophageal reflux disease on obstructive sleep apnea

**FIGURE 5:**
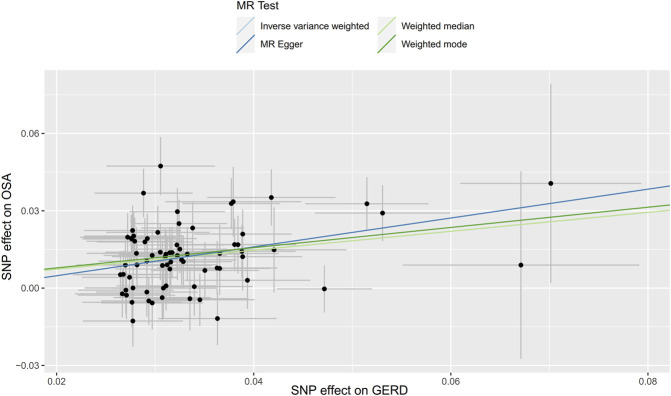
Scatter plot for the causal relationship of GERD on OSA. Abbreviation: MR, Mendelian randomization; SNP, single nucleotide polymorphism; GERD, gastroesophageal reflux disease; OSA, obstructive sleep apnea.

**FIGURE 6:**
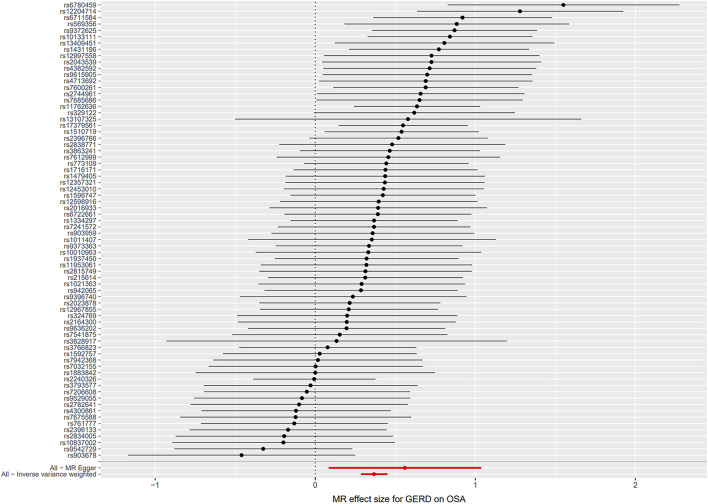
Forest plot for the causal relationship of GERD on OSA. Abbreviation: MR, Mendelian randomization; GERD, gastroesophageal reflux disease; OSA, obstructive sleep apnea.

Seventy-four independent SNPs strongly associated with GERD were selected as IVs in the MR analysis. The *F* statistics for these IVs were all larger than 10 (ranging from 207 to 669), indicating that they were strong IVs (Supplementary Tables S3). These SNPs are responsible for 3% of the population-level variation in GERD. After removing two SNP outliers detected by MR PRESSO (rs2734839, rs7527682), MR analysis was performed. The IVW results (OR: 1.44, 95% CI: 1.33–1.57, *p* = 7.74E-19), MR-Egger results (OR: 1.75, 95% CI: 1.09–2.81, *p* = 0.024), weighted median results (OR: 1.44, 95% CI: 1.30–1.61, *p* = 2.64E-11) and weighted mode results (OR: 1.48, 95% CI: 1.11–1.98, *p* = 0.010) all indicated that GERD was significantly associated with an increased risk of OSA (Figure 2B). The scatter plot and forest plot are shown in Figures 5, 6. Cochran’s Q test detected slight heterogeneity (Q = 91.75, *p* = 0.049). The MR-Egger intercept analysis did not reveal directional pleiotropy (*p* = 0.423) (Table 1). Moreover, the leave-one-out analysis indicated that no single SNP drove the results (Supplementary Figure S2).

## 4 Discussion

To the best of our knowledge, this is the first two-sample MR study to explore the causal association between OSA and GERD. According to our findings, OSA is causally related to GERD in individuals of European descent. Moreover, GERD also causally increases the risk of OSA. In view of the increasing health burden caused by OSA and GERD, this study provides new insight into the diagnosis and treatment of these two diseases.

Several case-control studies have failed to reveal a causal relationship between OSA and GERD due to the potential influence of confounders such as gender, age, obesity, physical activity, and alcohol use (Shibli et al., 2020). MR analysis can effectively avoid confounders and reverse causation bias in observational studies. It can also overcome the high cost and low practicability of randomized controlled trials. Thus, MR analysis is a powerful tool that can provide strong evidence that OSA is causally associated with GERD and that GERD is causally associated with OSA.

Results from a meta-analysis indicated that OSA and GERD are closely related, with a pooled OR of 1.75 (Wu et al., 2019). It is generally believed that OSA results in sleep fragmentation and sleep deprivation from awakenings (Yeghiazarians et al., 2021). A randomized controlled trial reported that sleep deficiency can increase oesophageal acid exposure, and even in healthy subjects, a lack of sleep can contribute to abnormal pH testing (Yamasaki et al., 2019). This might be the reason why OSA increases the incidence of GERD. An alternative hypothesis is that OSA can increase the respiratory workload and raise the abdominal-gastric pressure, which leads to lower oesophageal sphincter relaxations, and then reflux occurring (Tamanna et al., 2016; Ozcelik et al., 2017; Gleeson and McNicholas., 2022).

Previous cohort studies have revealed that more severe OSA symptoms are frequently encountered in patients with GERD, as compared to the healthy population (Emilsson et al., 2013; Kim et al., 2018). In addition, oesophagogastroduodenoscopy findings have demonstrated that GERD can cause OSA (Kim et al., 2018). These results illustrated that GERD might be associated with a higher risk of OSA, which is consistent with the current MR estimates. Thus, GERD may be a novel predictor of OSA diagnosis. The possible mechanism involves activation of the oesophageal-bronchial neuronal pathway due to reflux of the stomach contents, leading to bronchoconstriction (Houghton et al., 2016). However, the complex pathophysiology underlying the causal relationship between GERD and OSA is not completely understood. More research is required to further clarify this mechanism.

In the current MR analysis, the exposure and outcome genetic data were obtained from different European countries, which minimizes population stratification and sample overlap. Further, the GWAS summary data were obtained from large case-control samples (307,927 for OSA; 602,604 for GERD). Hence, this MR analysis is powered to detect genetic effects on outcome risks. Although a more liberal threshold (*p* < 5E-07) was used to select OSA-related IVs, the included genetic IVs were all strong IVs, with a minimum *F* statistic of 207. Non-etheless, this study has several limitations that should be acknowledged. First, the results of this study are based on data from individuals of European ancestry; thus, the findings may not apply to other ethnicities. Second, the *p* values for the Cochran Q test were less than 0.05, suggesting the presence of some heterogeneity in the effect estimates. However, this did not invalidate the random-effects IVW estimates, and the MR-Egger intercept analysis did not detect any directional pleiotropy, which provides validation of the robustness of the MR results.

Even though the mechanism underlying the association between OSA and GERD is not fully clear, the current MR analysis results suggest that OSA plays an important role in the pathogenesis of GERD, and *vice versa*. These findings have implications for reducing the risks of OSA and GERD. When managing and treating OSA patients, we must pay attention to their gastrointestinal symptoms. If we suspect that a patient has GERD symptoms (such as chest pain, chronic cough, laryngitis) (Gyawali et al., 2018), 24-h pH monitoring or upper gastrointestinal endoscopy is recommended. Non-pharmacologic and behavioural interventions for GERD patients (such as dietary control, weight reduction, and cutting alcohol consumption) may also be effective for OSA patients. In addition, patients with GERD can have latent OSA, so including questions about GERD when taking the patient’s clinical history may be significant in the screening and prevention of OSA.

## Data Availability

The original contributions presented in the study are included in the article/Supplementary Material, further inquiries can be directed to the corresponding author.
