# Supplementary material for: Causal association between obstructive sleep apnea and gastroesophageal reflux disease: A bidirectional two-sample Mendelian randomization study
Source: Front Genet. 2023 Apr 5;14:1111144. doi: 10.3389/fgene.2023.1111144 (PMC10113458; doi:10.3389/fgene.2023.1111144)
Supplement: Supplementary file 1 [file Table1.DOCX]

Supplementary Material

Causal association between obstructive sleep apnea and gastroesophageal reflux disease: A bidirectional two-sample mendelian randomization study

Qianyin Zhu, Lijiangshan Hua, Lingshan Chen, Tingyu Mu, Die Dong, Jiayi Xu, Cuizhen Shen*

*** Correspondence:** Cuizhen shen: shencuizhen@163.com

# Supplementary Figures


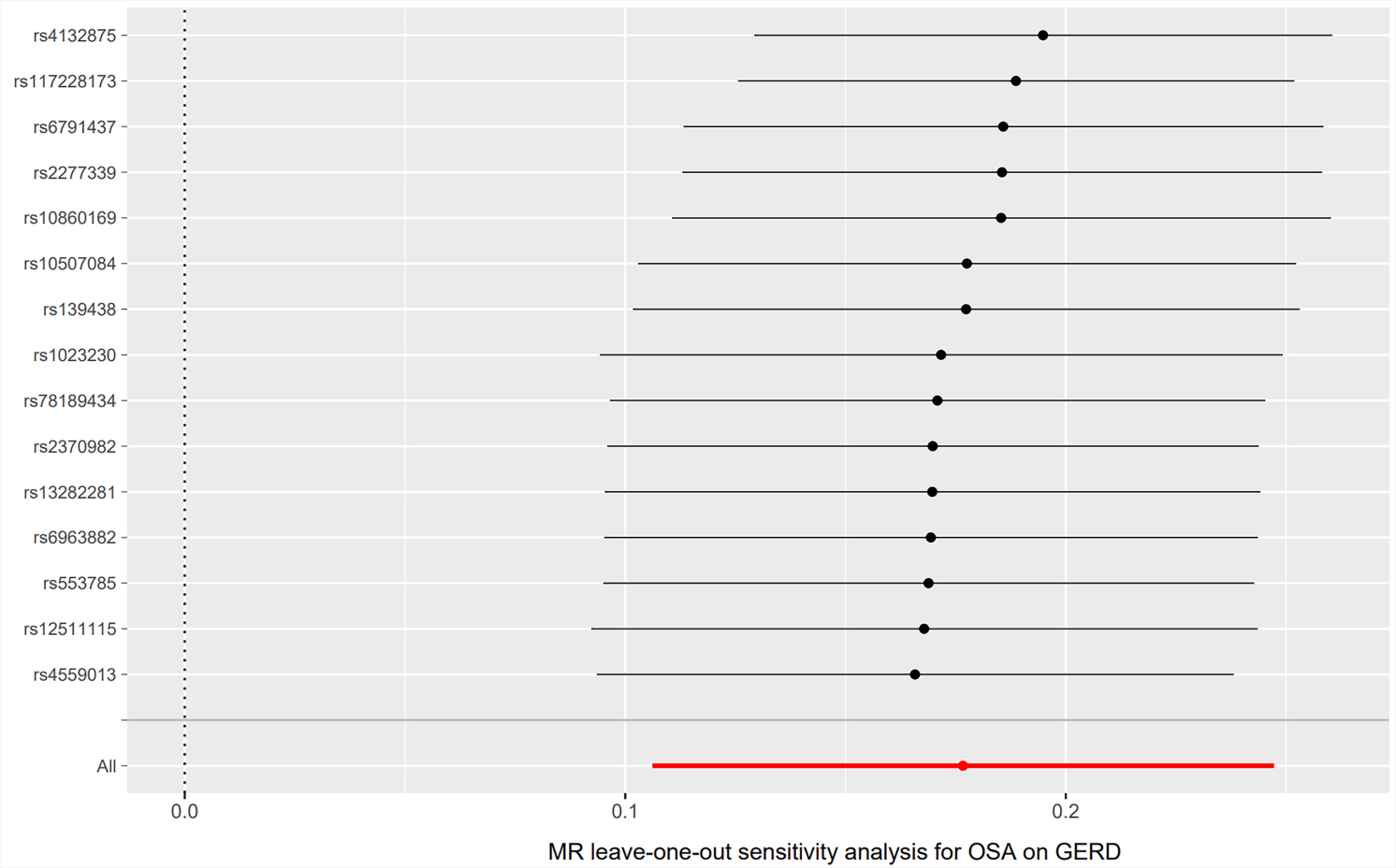


**Supplementary Figure 1.** MR leave-one-out sensitivity analysis for OSA on GERD. Abbreviation: MR, Mendelian randomization; OSA, obstructive sleep apnea; GERD, gastroesophageal reflux disease.


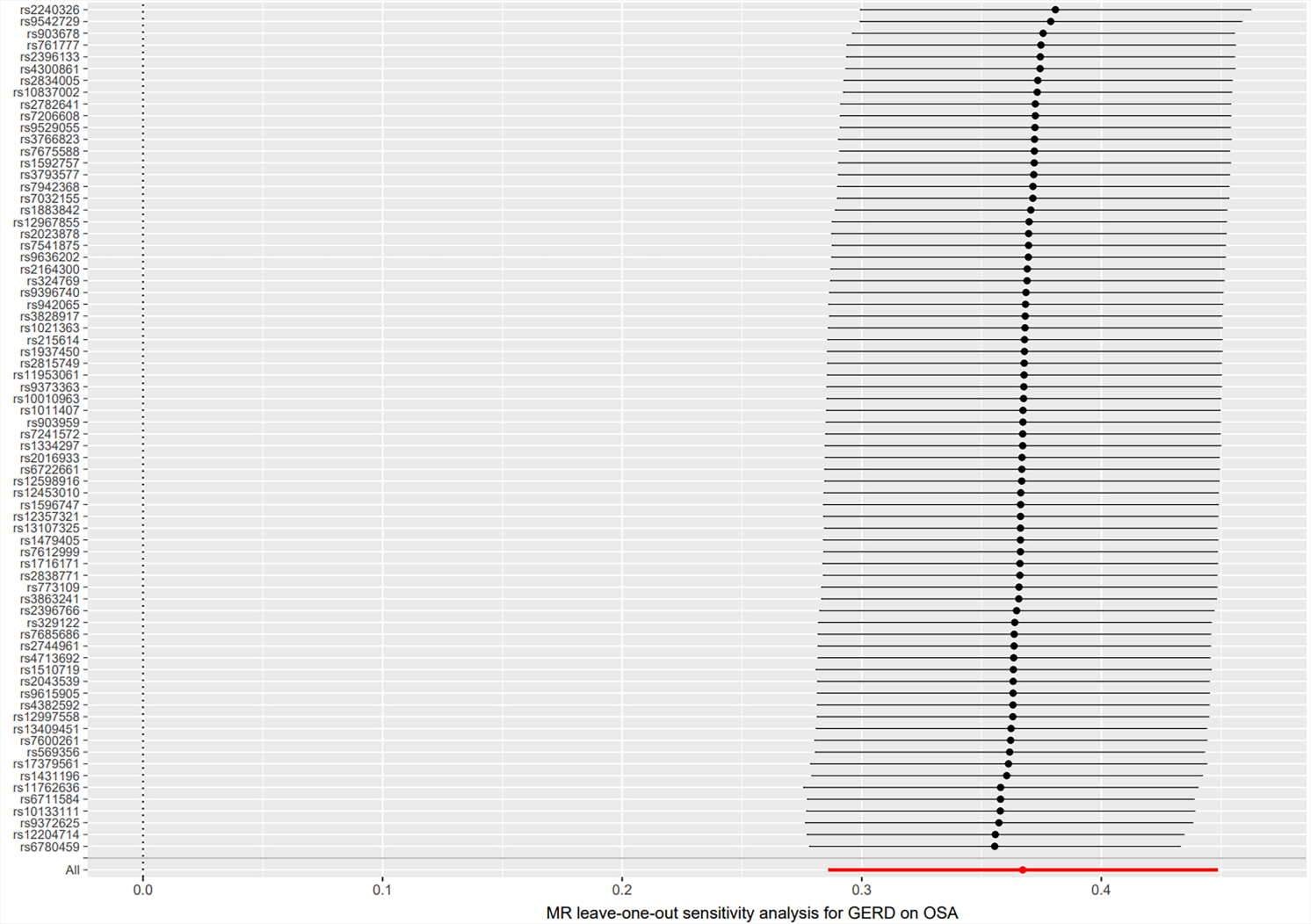


**Supplementary Figure 2.** MR leave-one-out sensitivity analysis for GERD on OSA. Abbreviation: MR, Mendelian randomization; GERD, gastroesophageal reflux disease; OSA, obstructive sleep apnea.

# Supplementary Tables

**Supplementary Table S1. Detailed information of studies and datasets used in the present study.**

| Exposure or outcome | Ancestry | Participants | GWAS ID | Web source |
| --- | --- | --- | --- | --- |
| Obstructive sleep apnea | European | 27,207 cases and 280,720 controls | finngen_R7_G6_SLEEPAPNO | https://r7.finngen.fi/pheno/G6_SLEEPAPNO |
| gastroesophageal reflux disease | European | 129,080 cases and 473,524 controls | ebi-a-GCST90000514 | https://gwas.mrcieu.ac.uk/datasets/ebi-a-GCST90000514/ |

Abbreviation: GWAS, Genome-wide association study.

**Supplementary Table S2. Detailed information of SNPs used in the MR analysis of OSA on GERD.**

| SNP | Chr:BP | Effect allele | Other allele | MAF | Exposure (OSA) | | |  | Outcome (GERD) | | | R^2^ | *F* statistic ^1^ |
| --- | --- | --- | --- | --- | --- | --- | --- | --- | --- | --- | --- | --- | --- |
|  |  |  |  |  | OR | 95% CI | *P*-value |  | OR | 95% CI | *P*-value |  |  |
| rs10125995 | 9:128145942 | T | C | 0.480 | 0.945 | 0.936-0.954 | 6.47E-10 |  | 1.016 | 1.011-1.021 | 0.001 | 0.0016 | 492.139 |
| rs1023230 | 13:20270158 | T | C | 0.922 | 0.916 | 0.901-0.931 | 1.36E-07 |  | 0.981 | 0.975-0.988 | 0.006 | 0.0011 | 343.441 |
| rs10507084 | 12:97753152 | T | C | 0.179 | 1.081 | 1.068-1.093 | 5.60E-11 |  | 1.012 | 1.000-1.023 | 0.242 | 0.0018 | 544.569 |
| rs10860169 | 12:97671568 | G | A | 0.290 | 0.947 | 0.937-0.957 | 8.29E-08 |  | 0.995 | 1.000-1.001 | 0.364 | 0.0012 | 378.463 |
| rs10917318 | 1:23168059 | T | C | 0.174 | 0.936 | 0.925-0.947 | 6.38E-08 |  | 1.011 | 1.000-1.017 | 0.070 | 0.0013 | 388.446 |
| rs11030323 | 11:28421065 | A | G | 0.477 | 1.049 | 1.039-1.058 | 1.99E-07 |  | 0.991 | 0.986-1.000 | 0.058 | 0.0011 | 346.998 |
| rs117228173 | 12:97878915 | C | T | 0.054 | 0.901 | 0.882-0.920 | 4.63E-07 |  | 1.026 | 1.000-1.043 | 0.127 | 0.0011 | 344.391 |
| rs12511115 | 4:47322972 | C | G | 0.295 | 1.060 | 1.049-1.070 | 6.82E-09 |  | 1.015 | 1.010-1.020 | 0.002 | 0.0014 | 432.788 |
| rs13282281 | 8:93297037 | A | C | 0.609 | 0.953 | 0.944-0.962 | 3.13E-07 |  | 0.987 | 0.986-0.992 | 0.007 | 0.0011 | 335.333 |
| rs139438 | 22:41605639 | T | C | 0.391 | 0.953 | 0.945-0.962 | 3.80E-07 |  | 0.992 | 0.987-1.000 | 0.111 | 0.0011 | 333.669 |
| rs2277339 | 12:57146069 | G | T | 0.129 | 0.932 | 0.919-0.945 | 3.44E-07 |  | 0.998 | 0.990-1.006 | 0.796 | 0.0011 | 347.509 |
| rs2370982 | 14:79890677 | T | C | 0.238 | 1.056 | 1.044-1.067 | 3.39E-07 |  | 1.016 | 1.010-1.022 | 0.009 | 0.0011 | 327.163 |
| rs2529273 | 7:74289929 | T | C | 0.780 | 0.942 | 0.931-0.952 | 5.67E-08 |  | 0.973 | 0.967-0.978 | 5.88E-07 | 0.0012 | 383.601 |
| rs4132875 | 16:73316054 | T | C | 0.624 | 1.050 | 1.040-1.060 | 2.51E-07 |  | 0.996 | 0.991-1.001 | 0.468 | 0.0011 | 344.026 |
| rs4559013 | 5:170851624 | G | A | 0.552 | 0.954 | 0.945-0.963 | 2.79E-07 |  | 0.985 | 0.980-0.989 | 0.001 | 0.0011 | 341.937 |
| rs553785 | 1:96895325 | A | G | 0.428 | 1.048 | 1.038-1.057 | 4.52E-07 |  | 1.014 | 1.009-1.018 | 0.005 | 0.0011 | 325.257 |
| rs6791437 | 3:168616977 | G | A | 0.409 | 0.950 | 0.941-0.959 | 3.11E-08 |  | 0.998 | 0.993-1.004 | 0.780 | 0.0013 | 398.081 |
| rs6963882 | 7:69293432 | G | C | 0.325 | 1.051 | 1.041-1.062 | 2.38E-07 |  | 1.014 | 1.009-1.019 | 0.007 | 0.0011 | 338.969 |
| rs742760 | 20:50985290 | T | A | 0.163 | 0.929 | 0.918-0.941 | 4.44E-09 |  | 0.970 | 0.964-0.976 | 1.28E-06 | 0.0015 | 452.435 |
| rs76229479 | 2:103123912 | C | A | 0.099 | 0.916 | 0.901-0.930 | 1.52E-08 |  | 1.009 | 1.000-1.016 | 0.214 | 0.0014 | 428.054 |
| rs78189434 | 9:126309260 | G | C | 0.101 | 1.080 | 1.064-1.096 | 2.54E-07 |  | 1.021 | 1.012-1.029 | 0.014 | 0.0011 | 330.098 |

Abbreviation: SNPs, single nucleotide polymorphisms; MR, Mendelian randomization; OSA, obstructive sleep apnea; GERD, gastroesophageal reflux disease; Chr:BP, chromosome: base-pair position (GRCh37); MAF, minor allele frequency; OR, odds ratio; 95% CI: 95% confidence interval.

^1^ *F*-statistic were calculated using the following formula: *R^2^*(N-2)/(1-*R^2^*), where *R^2^* is the proportion of variance in OSA explained by each instrument and N is the sample size of the GWAS for the SNP-OSA association.

**Supplementary Table S3. Detailed information of SNPs used in the MR analysis of GERD on OSA.**

| SNP | Chr:BP | Effect allele | Other allele | MAF | Exposure (GERD) | | |  | Outcome (OSA) | | | R^2^ | *F* statistic ^1^ |
| --- | --- | --- | --- | --- | --- | --- | --- | --- | --- | --- | --- | --- | --- |
|  |  |  |  |  | OR | 95% CI | *P*-value |  | OR | 95% CI | P-value |  |  |
| rs10010963 | 4:159839313 | T | C | 0.616 | 0.973 | 0.968-0.978 | 4.92E-08 |  | 0.991 | 0.981-1.000 | 0.355 | 0.0003 | 207.506 |
| rs1011407 | 2:60665768 | G | A | 0.122 | 0.959 | 0.951-0.965 | 1.09E-08 |  | 0.985 | 0.969-1.001 | 0.370 | 0.0004 | 227.883 |
| rs10133111 | 14:103377321 | A | G | 0.163 | 1.043 | 1.035-1.049 | 1.35E-10 |  | 1.036 | 1.024-1.047 | 0.001 | 0.0005 | 287.253 |
| rs1021363 | 10:106610839 | G | A | 0.642 | 0.969 | 0.964-0.974 | 5.10E-10 |  | 0.991 | 0.980-1.001 | 0.378 | 0.0004 | 270.060 |
| rs10837002 | 11:38565727 | G | C | 0.351 | 1.028 | 1.022-1.033 | 4.03E-08 |  | 0.995 | 0.984-1.004 | 0.574 | 0.0003 | 210.015 |
| rs11762636 | 7:2061111 | A | C | 0.180 | 0.950 | 0.943-0.955 | 1.88E-16 |  | 0.968 | 0.957-0.977 | 0.001 | 0.0008 | 472.433 |
| rs11953061 | 5:120144025 | T | C | 0.339 | 1.029 | 1.023-1.033 | 3.10E-08 |  | 1.009 | 0.999-1.018 | 0.341 | 0.0004 | 214.201 |
| rs12204714 | 6:152235339 | T | C | 0.632 | 0.972 | 0.966-0.976 | 7.92E-09 |  | 0.964 | 0.954-0.972 | 9.56E-05 | 0.0004 | 232.799 |
| rs12357321 | 10:21790476 | A | G | 0.311 | 1.032 | 1.026-1.037 | 1.33E-09 |  | 1.014 | 1.000-1.024 | 0.169 | 0.0004 | 259.925 |
| rs12453010 | 17:50316131 | T | C | 0.395 | 1.030 | 1.025-1.035 | 1.75E-09 |  | 1.013 | 1.000-1.022 | 0.179 | 0.0004 | 254.061 |
| rs12598916 | 16:60658751 | G | C | 0.275 | 0.967 | 0.962-0.972 | 6.87E-10 |  | 0.987 | 0.976-1.000 | 0.206 | 0.0004 | 265.831 |
| rs12967855 | 18:35138245 | G | A | 0.670 | 0.964 | 0.959-0.969 | 1.09E-12 |  | 0.992 | 0.982-1.002 | 0.460 | 0.0006 | 355.855 |
| rs12997558 | 2:41704580 | A | G | 0.359 | 1.028 | 1.023-1.033 | 3.04E-08 |  | 1.020 | 1.010-1.030 | 0.034 | 0.0004 | 214.634 |
| rs13107325 | 4:103188709 | T | C | 0.074 | 1.073 | 1.062-1.082 | 2.20E-14 |  | 1.041 | 1.000-1.082 | 0.293 | 0.0007 | 408.863 |
| rs1334297 | 13:58335375 | A | G | 0.734 | 0.962 | 0.956-0.967 | 1.14E-12 |  | 0.986 | 0.975-1.000 | 0.166 | 0.0006 | 354.211 |
| rs13409451 | 2:144257639 | G | A | 0.392 | 0.973 | 0.967-0.977 | 1.93E-08 |  | 0.978 | 0.968-0.987 | 0.021 | 0.0004 | 220.689 |
| rs1431196 | 18:50832102 | G | A | 0.428 | 1.033 | 1.027-1.037 | 2.65E-11 |  | 1.025 | 1.015-1.034 | 0.007 | 0.0005 | 310.350 |
| rs1479405 | 12:15387519 | T | C | 0.322 | 1.032 | 1.026-1.037 | 9.85E-10 |  | 1.014 | 1.000-1.024 | 0.168 | 0.0004 | 260.801 |
| rs1510719 | 4:140938116 | C | T | 0.383 | 0.962 | 0.957-0.966 | 3.84E-15 |  | 0.979 | 0.969-0.988 | 0.028 | 0.0007 | 431.116 |
| rs1592757 | 5:103889998 | C | G | 0.356 | 1.032 | 1.026-1.036 | 6.00E-10 |  | 1.001 | 0.991-1.010 | 0.928 | 0.0004 | 267.379 |
| rs1596747 | 2:193802478 | G | A | 0.494 | 1.032 | 1.026-1.036 | 1.00E-10 |  | 1.013 | 1.000-1.022 | 0.151 | 0.0005 | 291.277 |
| rs1716171 | 12:123716376 | T | C | 0.790 | 1.039 | 1.033-1.045 | 7.83E-11 |  | 1.017 | 1.000-1.028 | 0.133 | 0.0005 | 294.919 |
| rs17379561 | 1:98340139 | T | A | 0.144 | 1.055 | 1.047-1.061 | 1.08E-14 |  | 1.030 | 1.018-1.040 | 0.007 | 0.0007 | 419.662 |
| rs1883842 | 20:41223062 | G | T | 0.279 | 1.031 | 1.025-1.036 | 9.27E-09 |  | 1.000 | 1.000-1.011 | 1.000 | 0.0004 | 230.699 |
| rs1937450 | 1:66478840 | G | T | 0.538 | 1.032 | 1.027-1.037 | 7.07E-11 |  | 1.010 | 1.000-1.019 | 0.272 | 0.0005 | 299.008 |
| rs2016933 | 3:65653157 | G | C | 0.730 | 0.969 | 0.964-0.974 | 1.04E-08 |  | 0.988 | 0.977-1.000 | 0.256 | 0.0004 | 228.713 |
| rs2023878 | 19:18834124 | T | C | 0.192 | 0.964 | 0.958-0.970 | 3.04E-09 |  | 0.992 | 0.981-1.002 | 0.454 | 0.0004 | 246.629 |
| rs2043539 | 7:12253880 | A | G | 0.419 | 1.028 | 1.022-1.032 | 2.24E-08 |  | 1.020 | 1.010-1.029 | 0.037 | 0.0004 | 217.186 |
| rs215614 | 7:32347335 | A | G | 0.630 | 0.968 | 0.962-0.972 | 4.08E-11 |  | 0.990 | 0.979-1.000 | 0.312 | 0.0005 | 303.483 |
| rs2164300 | 4:67813017 | T | C | 0.523 | 0.974 | 0.969-0.978 | 4.13E-08 |  | 0.995 | 0.985-1.003 | 0.569 | 0.0003 | 210.807 |
| rs2240326 | 3:50128386 | A | G | 0.474 | 0.954 | 0.949-0.958 | 1.13E-22 |  | 1.000 | 0.991-1.009 | 0.973 | 0.0011 | 669.242 |
| rs2396133 | 7:109197067 | G | A | 0.475 | 1.030 | 1.024-1.034 | 1.11E-09 |  | 0.995 | 0.985-1.004 | 0.588 | 0.0004 | 259.110 |
| rs2396766 | 7:114318071 | A | G | 0.473 | 1.033 | 1.027-1.037 | 2.34E-11 |  | 1.017 | 1.000-1.026 | 0.067 | 0.0005 | 311.767 |
| rs2734839 | 11:113286490 | T | C | 0.607 | 0.972 | 0.967-0.976 | 8.79E-09 |  | 0.960 | 0.950-0.968 | 7.15E-06 | 0.0004 | 231.189 |
| rs2744961 | 6:34655000 | T | C | 0.358 | 1.030 | 1.024-1.034 | 5.81E-09 |  | 1.019 | 1.009-1.029 | 0.046 | 0.0004 | 236.412 |
| rs2782641 | 1:44013355 | A | G | 0.613 | 1.027 | 1.022-1.032 | 4.33E-08 |  | 0.997 | 0.987-1.006 | 0.770 | 0.0003 | 209.933 |
| rs2815749 | 1:72814783 | G | A | 0.801 | 1.040 | 1.033-1.045 | 1.07E-10 |  | 1.012 | 0.999-1.025 | 0.353 | 0.0005 | 290.521 |
| rs2834005 | 21:34291708 | C | T | 0.315 | 1.030 | 1.024-1.035 | 9.42E-09 |  | 0.994 | 0.984-1.004 | 0.574 | 0.0004 | 229.473 |
| rs2838771 | 21:46501576 | C | G | 0.647 | 0.972 | 0.967-0.977 | 2.91E-08 |  | 0.987 | 0.976-1.000 | 0.181 | 0.0004 | 217.478 |
| rs324769 | 12:83969240 | T | C | 0.449 | 0.974 | 0.968-0.978 | 3.05E-08 |  | 0.995 | 0.985-1.004 | 0.569 | 0.0004 | 213.766 |
| rs329122 | 5:133864599 | A | G | 0.420 | 0.971 | 0.966-0.976 | 3.05E-09 |  | 0.982 | 0.973-1.000 | 0.052 | 0.0004 | 246.147 |
| rs3766823 | 1:32197257 | A | G | 0.171 | 1.040 | 1.033-1.046 | 7.09E-10 |  | 1.003 | 0.992-1.014 | 0.784 | 0.0004 | 265.369 |
| rs3793577 | 9:23737627 | G | A | 0.538 | 1.027 | 1.022-1.032 | 2.49E-08 |  | 0.999 | 0.990-1.008 | 0.932 | 0.0004 | 218.941 |
| rs3828917 | 6:31465917 | T | G | 0.042 | 1.069 | 1.056-1.082 | 2.27E-08 |  | 1.009 | 0.972-1.046 | 0.806 | 0.0004 | 217.620 |
| rs3863241 | 8:73890335 | T | C | 0.527 | 1.033 | 1.028-1.038 | 1.49E-11 |  | 1.015 | 1.000-1.024 | 0.103 | 0.0005 | 317.456 |
| rs4300861 | 2:22549441 | T | C | 0.382 | 1.031 | 1.026-1.036 | 5.43E-10 |  | 0.996 | 0.987-1.005 | 0.689 | 0.0004 | 268.529 |
| rs4382592 | 9:134870755 | G | T | 0.700 | 0.970 | 0.965-0.975 | 8.20E-09 |  | 0.979 | 0.968-0.988 | 0.035 | 0.0004 | 232.169 |
| rs4713692 | 6:33807638 | T | C | 0.368 | 0.973 | 0.967-0.977 | 3.07E-08 |  | 0.981 | 0.971-0.990 | 0.042 | 0.0004 | 213.750 |
| rs569356 | 1:29136686 | G | A | 0.141 | 0.963 | 0.956-0.969 | 4.07E-08 |  | 0.967 | 0.954-0.980 | 0.013 | 0.0003 | 209.755 |
| rs6711584 | 2:104421692 | A | G | 0.452 | 1.033 | 1.027-1.037 | 2.66E-11 |  | 1.030 | 1.020-1.039 | 0.001 | 0.0005 | 310.733 |
| rs6722661 | 2:100806588 | A | G | 0.365 | 0.968 | 0.963-0.973 | 1.15E-10 |  | 0.987 | 0.978-1.000 | 0.188 | 0.0005 | 290.632 |
| rs6780459 | 3:104624105 | T | A | 0.747 | 1.031 | 1.025-1.036 | 3.14E-08 |  | 1.048 | 1.036-1.060 | 2.61E-05 | 0.0004 | 212.876 |
| rs7032155 | 9:122672771 | A | C | 0.592 | 1.028 | 1.023-1.033 | 1.63E-08 |  | 1.000 | 0.990-1.009 | 0.995 | 0.0004 | 224.274 |
| rs7206608 | 16:82872628 | G | C | 0.323 | 1.030 | 1.024-1.034 | 1.46E-08 |  | 0.998 | 0.988-1.008 | 0.872 | 0.0004 | 224.058 |
| rs7241572 | 18:77580712 | A | G | 0.209 | 1.037 | 1.031-1.043 | 9.49E-10 |  | 1.014 | 1.000-1.024 | 0.229 | 0.0004 | 266.398 |
| rs7527682 | 1:189172684 | G | A | 0.537 | 0.974 | 0.968-0.978 | 3.13E-08 |  | 1.028 | 1.018-1.037 | 0.004 | 0.0004 | 213.422 |
| rs7541875 | 1:190957589 | G | A | 0.426 | 1.028 | 1.022-1.032 | 1.61E-08 |  | 1.004 | 0.994-1.013 | 0.655 | 0.0004 | 221.295 |
| rs7600261 | 2:212622818 | T | C | 0.306 | 1.034 | 1.028-1.039 | 9.47E-11 |  | 1.024 | 1.013-1.033 | 0.019 | 0.0005 | 292.808 |
| rs7612999 | 3:35678337 | A | G | 0.245 | 1.031 | 1.025-1.036 | 4.90E-08 |  | 1.014 | 1.000-1.025 | 0.199 | 0.0003 | 207.962 |
| rs761777 | 10:134938075 | G | A | 0.254 | 1.035 | 1.029-1.040 | 4.71E-10 |  | 0.995 | 0.985-1.005 | 0.659 | 0.0005 | 272.498 |
| rs7675588 | 4:80734978 | A | C | 0.795 | 0.967 | 0.961-0.972 | 1.80E-08 |  | 1.004 | 0.991-1.016 | 0.738 | 0.0004 | 221.103 |
| rs7685686 | 4:3207142 | G | A | 0.422 | 0.972 | 0.967-0.977 | 1.14E-08 |  | 0.982 | 0.973-0.991 | 0.047 | 0.0004 | 229.331 |
| rs773109 | 12:56374695 | A | G | 0.335 | 0.963 | 0.957-0.967 | 8.71E-14 |  | 0.983 | 0.973-1.000 | 0.089 | 0.0006 | 389.273 |
| rs7942368 | 11:76465362 | T | C | 0.215 | 0.967 | 0.960-0.972 | 9.54E-09 |  | 0.999 | 0.988-1.010 | 0.959 | 0.0004 | 234.523 |
| rs903678 | 1:201809918 | A | G | 0.339 | 1.028 | 1.022-1.033 | 4.89E-08 |  | 0.987 | 0.977-1.000 | 0.202 | 0.0003 | 207.976 |
| rs903959 | 8:142630782 | A | T | 0.399 | 1.030 | 1.024-1.034 | 2.99E-09 |  | 1.011 | 1.000-1.020 | 0.266 | 0.0004 | 245.951 |
| rs9372625 | 6:98344031 | A | G | 0.383 | 0.963 | 0.958-0.967 | 2.62E-14 |  | 0.968 | 0.958-0.977 | 0.001 | 0.0007 | 405.657 |
| rs9373363 | 6:143150043 | G | A | 0.254 | 0.968 | 0.962-0.973 | 4.13E-09 |  | 0.989 | 0.979-1.000 | 0.258 | 0.0004 | 243.810 |
| rs9396740 | 6:17023108 | A | G | 0.249 | 0.969 | 0.963-0.974 | 1.47E-08 |  | 0.993 | 0.981-1.003 | 0.514 | 0.0004 | 223.485 |
| rs942065 | 14:94032065 | A | G | 0.634 | 1.031 | 1.026-1.036 | 8.45E-10 |  | 1.009 | 0.999-1.018 | 0.353 | 0.0004 | 264.339 |
| rs9529055 | 13:66957533 | A | G | 0.476 | 1.027 | 1.022-1.031 | 3.11E-08 |  | 0.998 | 0.988-1.006 | 0.809 | 0.0004 | 213.725 |
| rs9542729 | 13:31833578 | G | C | 0.202 | 0.964 | 0.958-0.970 | 1.41E-09 |  | 1.012 | 1.000-1.022 | 0.250 | 0.0004 | 256.793 |
| rs9615905 | 22:48875699 | T | C | 0.458 | 1.028 | 1.022-1.032 | 1.21E-08 |  | 1.019 | 1.010-1.028 | 0.036 | 0.0004 | 227.434 |
| rs9636202 | 19:18449238 | A | G | 0.267 | 0.966 | 0.960-0.970 | 1.51E-10 |  | 0.993 | 0.982-1.004 | 0.533 | 0.0005 | 289.553 |

Abbreviation: SNPs, single nucleotide polymorphisms; MR, Mendelian randomization; GERD, gastroesophageal reflux disease; OSA, obstructive sleep apnea; Chr:BP, chromosome: base-pair position (GRCh37); MAF, minor allele frequency; OR, odds ratio; 95% CI, 95% confidence interval.

^1^ *F*-statistic were calculated using the following formula: *R^2^*(N-2)/(1-*R^2^*), where *R^2^* is the proportion of variance in GERD explained by each instrument and N is the sample size of the GWAS for the SNP-GERD association.
